# Night target detection algorithm based on improved YOLOv7

**DOI:** 10.1038/s41598-024-66842-z

**Published:** 2024-07-09

**Authors:** Zheng Bowen, Lu Huacai, Zhu Shengbo, Chen Xinqiang, Xing Hongwei

**Affiliations:** Key Laboratory of Electric Drive and Control of Anhui Province, AnHui Polytechnic University, Wuhu, China

**Keywords:** YOLOv7, Square equalization, Gamma transform, GSConv module, Object detection, Characterization and analytical techniques, Imaging techniques

## Abstract

Aiming at the problems of error detection and missing detection in night target detection, this paper proposes a night target detection algorithm based on YOLOv7(You Only Look Once v7). The algorithm proposed in this paper preprocesses images by means of square equalization and Gamma transform. The GSConv(Group Separable Convolution) module is introduced to reduce the number of parameters and the amount of calculation to improve the detection effect. ShuffleNetv2_×1.5 is introduced as the feature extraction Network to reduce the number of Network parameters while maintaining high tracking accuracy. The hard-swish activation function is adopted to greatly reduce the delay cost. At last, Scylla Intersection over Union function is used instead of Efficient Intersection over Union function to optimize the loss function and improve the robustness. Experimental results demonstrate that the average detection accuracy of the proposed improved YOLOv7 model is 88.1%. It can effectively improve the detection accuracy and accuracy of night target detection.

## Introduction

With the construction of urban traffic roads, people travel more and more frequently at night. However, in some places with insufficient light or lack of light, accidents will occur frequently. Therefore, in the low-light environment at night, it is of great significance to accurately and quickly identify the road information ahead to reduce the incidence of traffic accidents.

In the past decade, with the enhancement of computer processing power, deep learning has developed rapidly and begun to be applied in many fields, and object detection is a field often studied by experts and scholars in many fields. Wang^1^ used Mosaic data enhancement method to detect high-density crowds, which can effectively improve the accuracy of crowd base. By adding CSP-OSA(Cross Stage Partial-One Shot Aggregation) structure to the backbone Network, Perveen^2^ improved the feature extraction capability of the Network, thus enhancing the robustness and real-time performance of the Network and improving the overall performance. Kumar^3^ introduced a bottom up PANet(Path Aggregation Network) structure to enhance feature fusion, which significantly improved the overall Network model's performance detection of small targets, not only improved the robustness of the overall Network, but also had better target resolution, which also reduced the detection speed. Gu^4^ proposed an improved target detection algorithm based on YOLOv5. By using a more complex Network structure and more accurate loss function, traffic detection in complex scenarios was not only realized, but also the detection speed was slightly improved. Guan^5^ dynamically enhanced the original Network by adding the improved Retienx algorithm and improved the training effect of the model by replacing the Focus layer with the CBS (Conflict Based Search) layer, to improve the accuracy of night target detection. Cao^6^ added the SimAM(Simple Attention Mechanism) based on YOLOv7-tiny to make the Network focus on the region of interest, which improved the accuracy, robustness, and generalization ability. Rio^7^ introduced ShuffleNet Network structure based on YOLOv7-tiny to improve the detection accuracy and introduced GSConv module to reduce the number of parameters and calculation amount of the model, to improve the detection effect.

Even with the continuous improvement of target detection technology, there are still some problems in this technology. Especially in the night detection, there are still problems of wrong detection and missing detection. Therefore, based on YOLOv7, this paper introduces GSConv module to reduce the number of parameters and calculation amount in the training process, and selects ShuffleNetv2_ × 1.5 as the feature extraction Network. The overall structure of the Network can have high tracking accuracy while reducing the number of parameters. The hard-swish activation function is adopted to greatly reduce the delay cost, and the EIoU function is replaced by SIoU function, to optimize the loss function and improve the robustness and generalization ability. In this paper, experiments are carried out on self-made data sets, and the results show that the improved algorithm has better detection results and practical significance in night target detection.

## YOLOv7 algorithm

**Figure 1:**
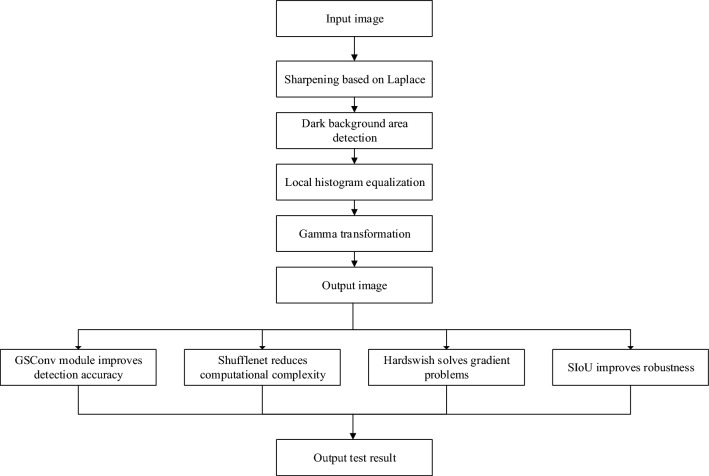
Object detection flow chart.

The methods for target recognition and detection mainly include traditional methods and deep learning-based methods. The traditional detection method is to obtain the output result by combining the feature extraction algorithm and the machine learning algorithm of the classifier. The detection method based on deep learning is divided into two-stage and one-stage. Although two-stage can meet the detection requirements and is stronger than one-stage in detection accuracy, its detection speed is inferior to one-stage, which also makes it not applicable to mobile terminals. Therefore, this paper chooses one-stage which has more research value. The one-stage target detection algorithm can detect the target faster by simplifying the process of candidate box. The main flow chart for this article is shown as Fig. 1.

**Figure 2:**
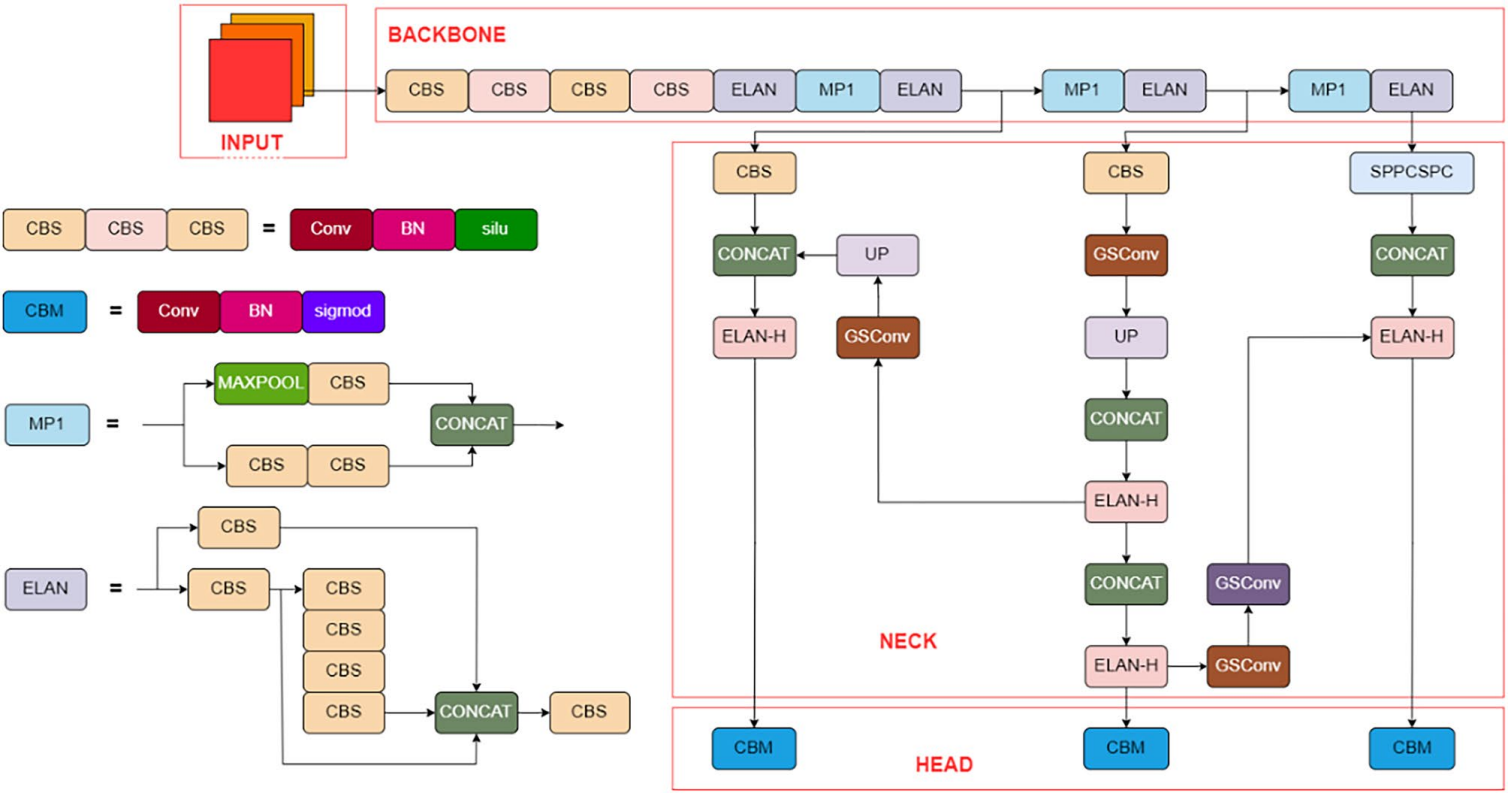
The network structure of the algorithm in this paper.

In 2014, Girshick R^8^ proposed R-CNN(Region Convolutional Neural Network), and object detection based on deep learning began to replace traditional detection algorithms. In 2016, Redmon J^9^ proposed the YOLOv1 algorithm with a single Network structure, which transformed the target detection task into a regression problem. By dividing the input image into multiple grids, each grid was used to identify the category and boundary box of the predicted target. Although YOLOv1 has a fast detection speed, However, its detection effect on small targets and overlapping targets is not good. In view of the low detection accuracy of YOLOv1, Redmon J^10^ proposed the YOLOv2 algorithm in 2017. Based on YOLOv1, the depth and multi-scale prediction of Network structure were improved, so that the multi-scale prediction of the algorithm allowed the Network to detect targets at different scales. Therefore, compared with the YOLOv1 algorithm, the improved YOLOv2 algorithm has significantly improved the detection algorithm of small targets. In addition, the YOLOv2 algorithm also adopts anchor boxes technology, which can predict the location and size of targets more accurately. Despite this, YOLOv2 still has some problems, such as low recall rate of small target detection and poor effect of intensive target detection. To solve the problem of poor detection effect of YOLOv2 on small targets, Redmon^11^ proposed YOLOv3 based on YOLOv2. By introducing cross-layer addition operation of residual Network resent, the detection accuracy of small targets was improved, but there were still problems such as low detection recall rate and poor detection effect of dense targets. Bochkovskiy^12^ proposed the YOLOv4 algorithm in 2020, using CSP-Darknet(Cross Stage Partial Darknet) instead of Darknet to increase the receptive field, which improved the detection accuracy of YOLOv4, but its training and prediction time increased and it lacked generalization. In the same year, Jocher^13^ proposed YOLOv5, which has better detection speed compared with previous versions of YOLO series. Aiming at the problem of the increase of computation amount of YOLO series models and the serious imbalance of positive and negative samples. Li^14^ proposed YOLOv6 based on YOLOv3, which reduces the calculation amount of the model by eliminating the anchor frame, to achieve the imbalance of positive and negative samples, and further improve the detection accuracy. In the same year, Choi^15^ proposed YOLOv7, which adopted the data enhancement method in the input stage to enable mobile devices to maintain the detection speed while having good detection accuracy. The improved structure of YOLOv7 in this paper is shown in Fig. 2.

BACKBONE: First, after four convolutional layers, the feature map becomes 160 * 160 * 128. Then it will go through the ELAN module, ELAN is composed of a few CBS, the size of its input and output characteristics remains unchanged, the number of channels will change in the first two CBS, the following several input channels are consistent with the output channel, and the last CBS output is the required channel.

NECK: First, for BACKBONE's final output of 32 times down sampling feature diagram C5, and then through the SPPCSPC, the number of channels from 1024 to 512. First, according to top down and C4, C3 fusion, P3, P4 and P5; Then press bottom-up to merge with P4 and P5. This is basically the same as YOLOv5, the difference is that the CSP module in YOLOv5 is replaced by the ELAN-H module, and the down sampling is changed to MP2 layer.

HEAD: The main body of this module is the REP module, which is divided into two categories, namely the training module train and the inference module deploy. The training module has three branches, which are 3 × 3 convolution for feature extraction, 1 × 1 convolution for smoothing features, and identity without convolution operation. Finally, the three branches are added. The inference module consists of a 3 × 3 convolution, stride 1, which is derived from the reparameterization of the training model.

## Algorithm improvement

### Image enhancement algorithm

**Figure 3:**
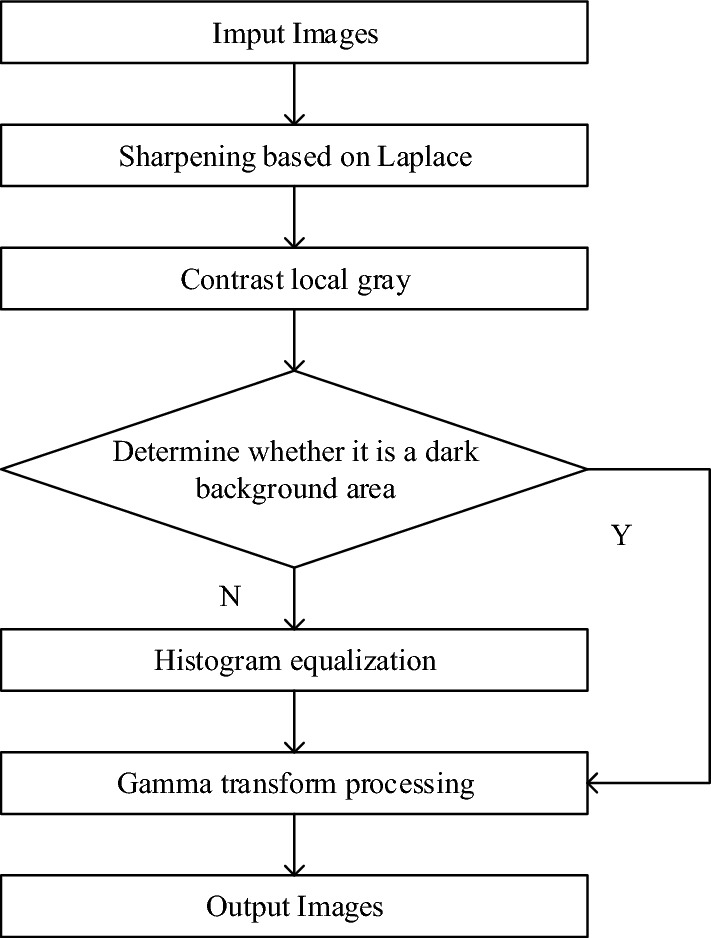
Algorithm flow chart of this paper.

For the blurring and unharness of the night image, this paper uses the sharpening method based on Laplace operator to process the image, judge the dark background area by comparing the local gray level, and carry out square equalization and Gamma transformation processing for the local dark background area, to improve the clarity of the image. The main flow of the algorithm is shown in Fig.3.

#### Laplacian operator

Laplacian operator is a simple isotropic differential operator, whose rotation invariance makes it convenient to calculate gray values in images. Laplacian operator is shown in formula (1):

1 $$\nabla^{2} f\left( {x,y} \right) = \frac{{\partial^{2} f}}{{\partial x^{2} }} + \frac{{\partial^{2} f}}{{\partial y^{2} }}$$

where, $f\left( {x,y} \right)$ is the gray value of the point $\left( {x,y} \right)$ in the image。

#### Dark background area detection

In the nighttime environment, it is easy for human beings to ignore some important factors in the environment, whether by the naked eye or by using the ordinary front-facing camera of the vehicle, and it is often because of ignoring these factors that accidents will occur. Therefore, this section will identify the dark background area in the collected images. Thus, the recognized images that are not easily detected are Gamma transformed.

Suppose there is an image with size *N* and gray level *L*, and the number of pixels with gray level is represented, then the normalized histogram *H* of gray level is defined as follows:

2 $$\begin{array}{*{20}l} {H\left( {r_{i} } \right) = \frac{{n_{i} }}{N}} & {i = 0,1, \cdots ,L - 1} \\ \end{array}$$

By comparing the local gray mean variance with the global gray mean variance, the dark regions of the night image can be detected.

#### Local histogram equalization

Local histogram equalization evolved from global histogram equalization. Different from global balanced histogram, it no longer operates on the entire human image, but divides the image into several small local blocks, and then performs histogram equalization operations on each small local block, and finally recombines all the equalized small local blocks into the final output image required.

The conversion function of the local equalization histogram is defined by the following formula:

3 $$\begin{array}{*{20}l} {T\left( {r_{i} } \right) = \sum\limits_{j = 0}^{i} {H\left( {r_{i} } \right)} = \sum\limits_{j = 0}^{i} {\frac{{n_{j} }}{N}} } & {\left\{ {\begin{array}{*{20}l} {i = 0,1, \cdots ,L - 1} \\ {0 \le r_{i} \le 1} \\ \end{array} } \right.} \\ \end{array}$$

Its advantage is that it can enhance the local details of the image, to avoid the problem of excessive enhancement or excessive distortion caused by global histogram equalization.

#### Gamma transformation

**Figure 4:**
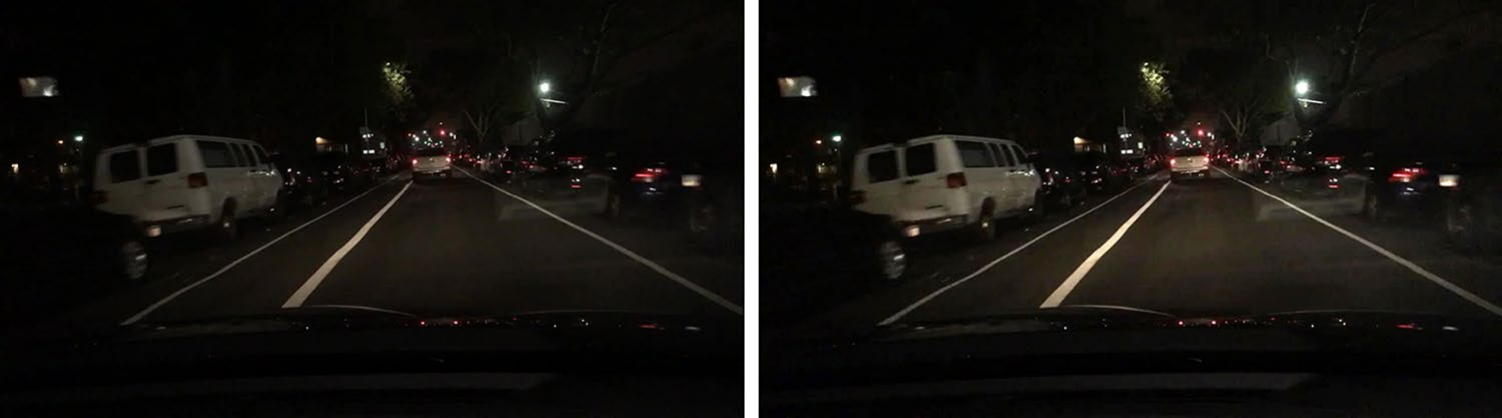
The results before and after processing.

Gamma transform, also known as power law, transform, is a nonlinear image enhancement method, which is mainly used to correct the overall image and soften the brightness of the image. In the nighttime environment, the images collected by the mobile terminal are mostly in low brightness and cannot be well recognized by human eyes, so it is necessary to Gamma transform the images collected first. Gamma transform is a nonlinear operation on the gray value of the input image, so that the gray value of the output image is exponentially related to the input. The formula is as follows:

4 $$V_{out} = AV_{in}^{\gamma }$$

where, $V_{out}$ is the output gray value, $V_{in}$ is the input gray value, $A$ is the gray scale factor, $\gamma$ is the gamma factor size. After Gamma transformation, dark detail and light detail will change correspondingly. As shown in the Fig. 4, when Gamma < 1, dark detail will be enhanced while light detail will be compressed; When Gamma > 1, light detail is enhanced while dark detail is compressed, When Gamma = 1 is the original image.

The comparison between the original image and the Gamma transformed image is shown in Fig. 4. The images processed by Gamma nonlinear transform have richer colors, which is conducive to the detection and recognition of later images.

### GSConv module

**Figure 5:**
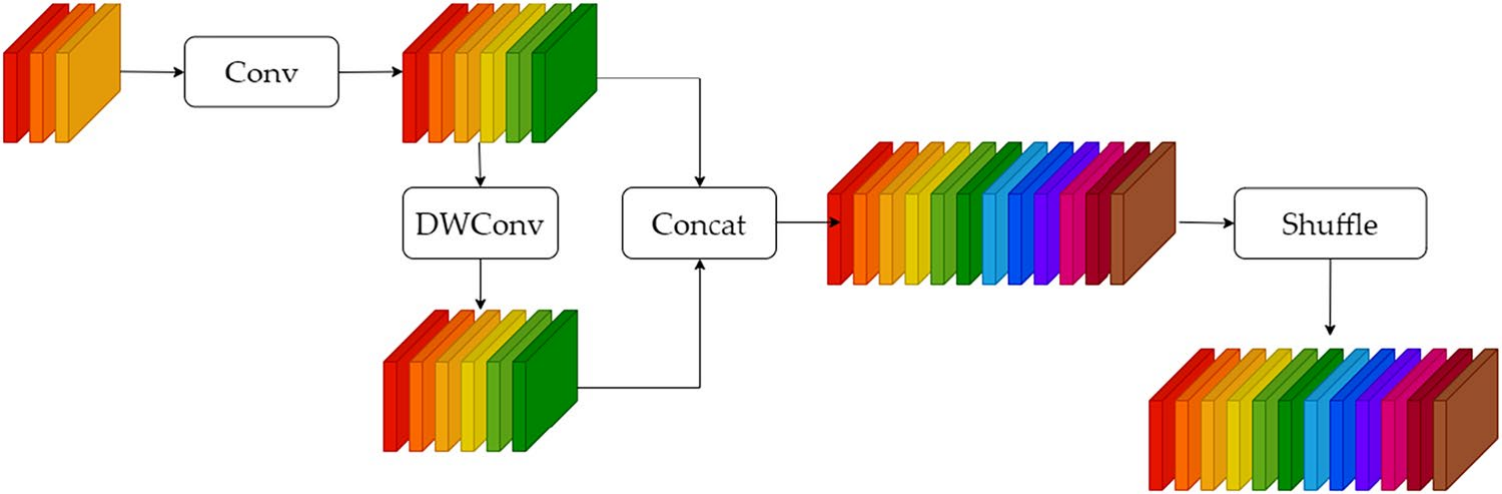
GSConv module.

GSConv, as a variant of convolutional neural Network, adopts adaptive graph structure to carry out image segmentation and image annotation tasks. Compared with traditional GCN (Graph Convolutional Network), GSConv learns graph structure to capture context information in images, so it can better adapt to the requirements of different image tasks and achieve better performance. As a combination of DWConv(Depth Wise-separable Convolution), SConv (Standard Convolution), and Shuffle modules, it not only effectively utilizes the computing power of DWConv module, but also has the detection accuracy of SConv module. Its structure is shown in Fig. 5.

It can be seen from the Fig. 5 above that GSConv first undersamples an ordinary convolution, then uses DWConv deep convolution, and concatenates the results of SConv and DSConv, and finally performs shuffle operation, so that the corresponding channel numbers of the previous two convolution are next to each other. The main purpose is to make the output of DSConv as close as possible to SConv. The module uses shuffle to infiltrate the information generated by SConv through intensive convolution operation into each part of the information generated by DSConv, which can greatly reduce the negative impact of DSConv defects on the model, and make it play the advantages of DSConv, that is, occupy less memory and speed up calculation.

### Shufflenet

**Figure 6:**
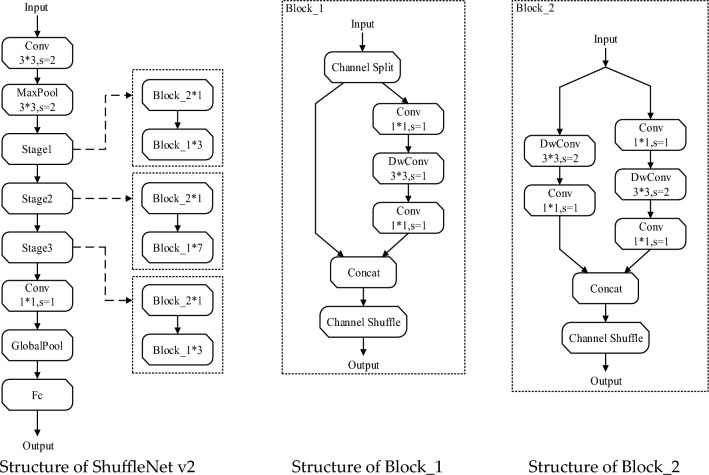
Structure of ShuffleNetv2 model.

Shufflenet Network structure is composed of Group Convolution and depth-wise separable Convolutions, which not only effectively reduces the computational complexity, but also enhances the depth of the overall Network structure, thus enhancing the extraction of image features. ShuffleNetv2 is a lightweight Network based on ShuffleNetv1 and MobileNetv2, which has the advantages of high precision and high speed. Its structure is shown in Fig. 6.

ShuffleNetv2 consists of a convolutional layer, a pooling layer, three stages, a global average pooling, and the final fully connected layer. Stages 1 to 3 are formed by stacking two different residual blocks, Block_1 and Block_2, and subsequently using the SoftMax function on the fully connected layer to output a 128-dimensional feature vector. At the beginning of each unit, the channel is divided into two branches, one branch is used for identity mapping, and the other branch is convolved by multiple layers to ensure that the number of input channels is the same as the number of output channels. Different from ShuffleNetv1, ShuffleNetv2's 1 × 11 ×  11 × 1 group convolution is not used again. The two branches end up using the channel concatenate operation instead of Channel Shuffel. According to the number of output channels of each layer of the Network, ShuffleNetv2 can be divided into four models of different sizes: ShuffleNetv2 × 0.5, ShuffleNetv2 × 1.0, ShuffleNetv2 × 1.5, and ShuffleNetv2 × 2.0. The ShuffleNetv2 × 0.5 model is the smallest, and ShuffleNetv2 × 2.0 model is the largest. Considering the depth and feature extraction capabilities of the Network, we selected ShuffleNetv2 × 1.5 as the feature extraction Network of DeepSORT in our study.

### Selection of activation function

**Figure 7:**
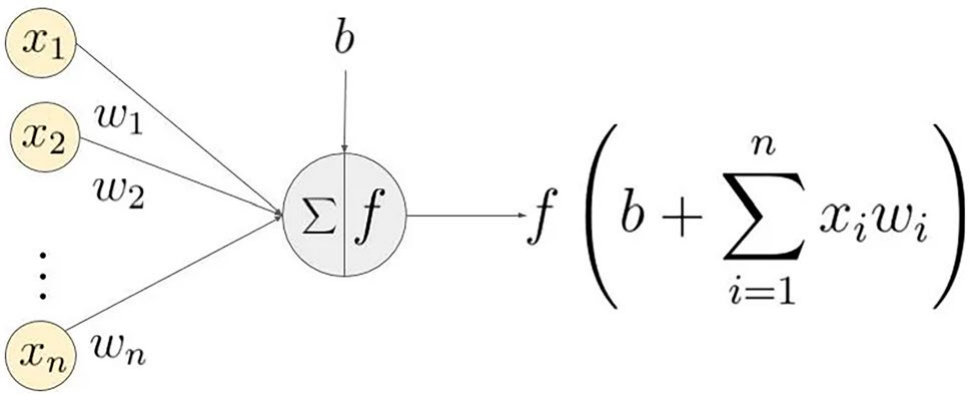
Activate function structure diagram.

As a function running on the neurons of a neural Network, the activation function is mainly responsible for mapping the input to the output of the neuron. Due to the nonlinear, differentiable, monotone, and other properties of the activation function, they can introduce nonlinear characteristics into the Network, which also makes the activation function play a very important role in the neural Network. The general structure of the activation function is shown in Fig. 7.

The Hard-Swish activation function replaces the ReLU(Rectified Linear Unit) activation function with the traditional Swish activation function, and its formula is as follows:

5 $$HardSwish\left( x \right) = \left\{ {\begin{array}{*{20}l} {0,} & {x \le - 3} \\ {\frac{1}{6}x^{2} + \frac{1}{2}x} & { - 3 < x < 3} \\ {x,} & {x \ge 3} \\ \end{array} } \right.$$

The derivation of HardSwish activation function is simple, and it can effectively prevent the saturation phenomenon caused by gradient gradually approaching zero during training, and further improve the expression ability of the Network model.

### Selection of loss function

**Figure 8:**
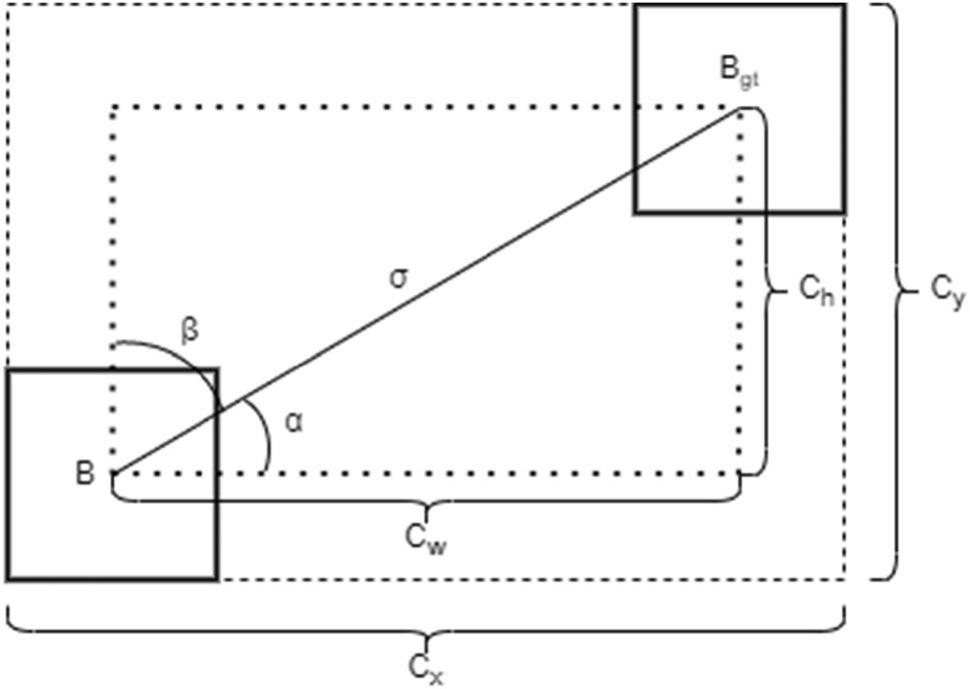
SIoU loss function.

As the prediction error of the model, it is often used to measure the difference between the predicted results of the model and the actual label. In supervised learning tasks, the loss function usually calculates the error by comparing the predicted results of the model with the real label. Hou Zhiqiang^16^ et al. proposed GIoU, that is, adding the minimum external rectangle of the prediction box and the real box. However, when the prediction box and the real box are in the included state, the GIoU loss function cannot determine the position relationship between the two boxes. Zheng et al.^17^ proposed the DIoU loss function. By introducing the minimum external rectangle, the penalty term that maximizes the overlapping area of the rectangle becomes the standardized distance between the center point of the minimum prediction box and the real box, to shorten the time of the loss convergence process, but the aspect ratio is not considered in this loss function. The CIoU proposed by Wang Yongshun^18^ et al. further optimized the regression accuracy of the model, but there was some ambiguity in the weight design of the aspect ratio, and the balance problem of sample difficulty was not considered. Zhang^19^ et al proposed EIoU loss function based on GIoU. By calculating overlap loss, center distance loss and width and height loss, EIoU solved the errors of GIoU loss function in horizontal and vertical directions and improved the speed of convergence and the accuracy of regression. However, it would consume a lot of time in the training of large-scale data. And the processing of different target scales is not flexible enough. Li Gong^20^ et al proposed SIoU based on CIoU. By treating the parameters of the boundary box for regression, the computational positioning loss was reduced, and the convergence speed of the model was accelerated without increasing the complexity of the network structure. SIoU loss function is a loss function composed of Angle loss, distance loss and shape loss. Due to its good accuracy and robustness, SIoU loss function is chosen in this paper. SIoU loss function is shown in Fig. 8.

Where, represents the center point of the prediction box, represents the center point of the real box, represents the distance from the center point of the real box to the center point of the prediction box, and is the width and height of the minimum external rectangle of the real box and the prediction box, and is the height difference and width difference of the center point of the real box and the prediction box, and is the balance parameter.

## Experimental results and analysis

The data sets used in this paper are all homemade night target data sets, about 40% of them were taken from the VOC2012 dataset^21^, and the rest were collected by me through filming and other means. which were divided into training sets, verification sets and test sets according to the ratio of 7:2:1. Among them, the number of training sets was 910, the number of verification sets was 260 and the number of test sets was 130. This paper conducted several experiments.

**Figure 9:**
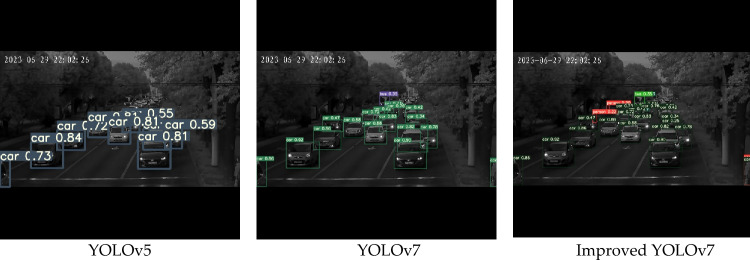
Effect comparison chart in bright environment.

As can be seen from the Fig. 9, compared with original YOLOv5 algorithm and original YOLOv7 algorithm, improved YOLOv7 algorithm can more effectively prevent the occurrence of problems such as missed detection and wrong detection. Therefore, the improved algorithm not only had better detection accuracy, but also was suitable for small target detection scenarios. Compared with the original YOLOv5 algorithm and original YOLOv7 algorithm, the improved YOLOv7 algorithm displayed superior performance. The precision level AP50 was improved, and the model converged more rapidly. And the results show that compared with YOLOv5 and YOLOv7, the improved algorithm in this paper had improved 4.9% and 5.6% in Precision P, 6.5% and 6.1% in Recall R, and 9.3% and 8.5% in mAP@.5, respectively. mAP@.95 increased by 4.0% and 4.7% respectively, indicating that the improved method in this paper not only maintains high detection accuracy, but also maintains good stability. Therefore, the improved algorithm in this paper was suitable for night target detection scenarios.

By comparing original YOLOv5 algorithm, original YOLOv7 algorithm and the improved YOLOv7 algorithm in this paper, the analysis of experimental results was shown in Table 1:

**Table 1:** Analysis of experimental results.

Network model	P(%)	R(%)	mAP@0.5(%)	mAP@0.5:0.95(%)
YOLOv5	80.3	76.8	77.8	44.3
YOLOv7	84.6	77.2	79.6	43.6
Improved YOLOv7	90.2	83.3	88.1	48.3

**Figure 10:**
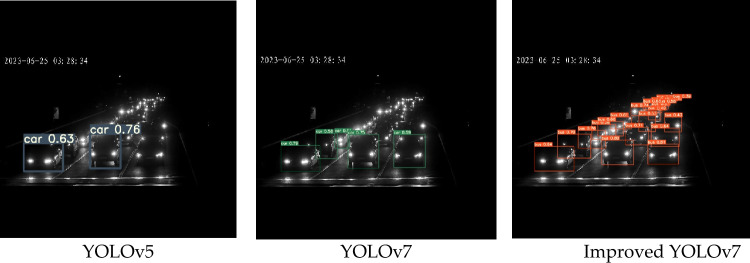
Effect comparison chart in dark environment.

The detection effects in dark environment of the original YOLOv5 algorithm, original YOLOv7 algorithm and the improved YOLOv7 algorithm in this paper were shown in Fig. 10. As can be seen from Fig. 10, the detection effect of original YOLOv5 algorithm and original YOLOv7 algorithm was not good in dark environment, in which there are a lot of missed detection, while improved YOLOv7 algorithm basically solved the problem of missed detection in dark environment.

The improved YOLOv7 algorithm proposed in this study used ShuffleNetv2 × 1.5 as the night target detection feature extraction Network. To verify the effectiveness of the improved algorithm, we conducted the ablation experiment on the homemade dataset. The input image size of the Network was 640*640, other parameters remaining unaltered, and the training epoch was set to 300. The results of the ablation experiment were shown in Table 2:

**Table 2:** Ablation experiment of ShuffleNetv2  ×  1.5.

Network model	P(%)	R(%)	mAP@0.5(%)	mAP@0.5:0.95(%)
YOLOv7	84.6	77.2	79.6	43.6
YOLOv7 + ShuffleNetv2 × 1.5	86.1	78.5	82.9	44.3

The purpose of ablation experiments is to remove a specific part of the system and to study the effect of that part on the system within a controlled variable manner. If the performance of the system is not greatly lost after the removal of this part, then this part is not of great importance to the whole system; If the system performance is significantly reduced after removal, it indicates that this part of the design is essential. This paper compared the ablation experiments of YOLOv5 and improved YOLOv7, including the use of various loss functions to complete the ablation experiments. The results of ablation experiment were shown in Table 3. As can be seen from the experimental results in the Table 3, when YOLOv5 adopts different loss functions CIoU, EIoU and SIoU, mAP@.5 was 77.8%, 80.1% and 81.9% respectively; when YOLOv7 adopts EIoU loss function, mAP@.5 was 79.6%. Compared with other algorithms, the improved YOLOv7 algorithm in this paper improved 10.3%, 8%, 6.2% and 9.5% respectively on mAP@.5.

**Table 3:** Ablation experiment of loss function.

Network model	CIoU	EIoU	SIoU	mAP@0.5(%)
YOLOv5	√			77.8
YOLOv5		√		80.1
YOLOv5			√	81.9
YOLOv7		√		79.6
Improved YOLOv7			**√**	88.1

As can be seen from the experimental results in the Table 4, when detecting images that were not enhanced by the algorithm, the detection accuracy used GSConv module was 5.7% higher than that without GSConv module, and when detecting images that were enhanced by the algorithm, the detection accuracy used GSConv module was 6.5% higher than that without GSConv module. When we do not used GSConv module, the image enhancement algorithm can improve the detection accuracy by 3.1%, while when we used GSConv module, the image enhancement algorithm can improve the detection accuracy by 3.9%. Both the image enhancement algorithm and GSConv module can improved the accuracy of vehicle detection at night.

**Table 4:** Ablation experiment of image enhancement and GSConv.

Network model	image enhancement	adding GSConv	P	R	mAP@0.5(%)	mAP@0.95(%)
YOLOv7			83.9	77.1	78.5	42.8
YOLOv7		√	87.6	85.2	84.2	45.6
YOLOv7	√		84.5	79.2	81.6	43.8
Improved YOLOv7	√	√	90.2	83.3	88.1	48.3

## Conclusion

In this paper, an improved YOLOv7 night target detection. By imported the GSConv module of YOLOv7,selected ShuffleNetv2 × 1.5, adopted the hard-swish activation function and using SIoU function to take the place of the EIoU function. Experimental results demonstrated that the average detection accuracy of the proposed improved YOLOv7 model was 88.1%. It can effectively improve the detection accuracy and accuracy of night target detection, At the same time, the error detection of moving target was reduced in the actual detection process, which proves the practicality and effectiveness of the improved algorithm. This also made the improved algorithm in this paper had better effect on target detection at night.

## Data Availability

The datasets used and/or analysed during the current study available from the corresponding author on reasonable request. All other data supporting the main conclusions of this study can be found in the main text.
